# Association between Sarcopenia and Renal Function in Patients with Diabetes: A Systematic Review and Meta-Analysis

**DOI:** 10.1155/2019/1365189

**Published:** 2019-11-18

**Authors:** Satoshi Ida, Ryutaro Kaneko, Kanako Imataka, Kazuya Murata

**Affiliations:** Department of Diabetes and Metabolism, Ise Red Cross Hospital, Mie 516-8512, Japan

## Abstract

Previous studies involving patients with diabetes have indicated that sarcopenia is related to renal function. The objective of the present study was to investigate the association between sarcopenia and urinary albumin level, urinary protein level, and estimated glomerular filtration rate (eGFR) in patients with diabetes. A meta-analysis of observational studies was conducted. A literature search was performed using MEDLINE, Cochrane Controlled Trials Registry, and ClinicalTrials.gov. Data were extracted from studies investigating the association between sarcopenia and urinary albumin level, urinary protein level, and eGFR and by calculating odds ratio (OR) and 95% confidence intervals (CIs). Statistical analysis was performed using a random-effects model to calculate pooled OR and 95% CI. Six studies (2662 patients) that met the criteria were included in the meta-analysis. Sarcopenia was significantly associated with urinary albumin level with a pooled OR of 2.11 (95% CI, 1.55–2.88; *P* < 0.001). The pooled ORs of the associations between sarcopenia and urinary protein level and decreased eGFR were 1.82 (95% CI, 1.13–2.92; *P* = 0.01) and 3.75 (95% CI, 1.24–11.41), respectively. Sarcopenia was significantly associated with urinary albumin level, urinary protein level, and decreased eGFR. However, further investigations are needed, including meta-analyses with a larger number of studies.

## 1. Introduction

Sarcopenia, a condition that is characterized by the loss of skeletal muscle mass [1], has received increased attention in recent years. The loss of skeletal muscle mass begins in the 30s with a decrease of 3%–5% every 10 years [2]. This loss is accelerated in elderly individuals [2, 3]. Sarcopenia is associated with a decrease in the activities of daily living, a decrease in quality of life, and cardiovascular diseases [4–6]. Previous studies have shown that the frequency of sarcopenia is higher in patients with diabetes than that in patients without diabetes [7]. This suggests that chronic inflammation, oxidative stress, and insulin resistance play a role in the onset of sarcopenia [8, 9]. Thus, caution is advised in patients with diabetes to prevent sarcopenia.

Chronic kidney diseases in patients with diabetes lead to end-stage renal failure, which is closely associated with the onset of cardiovascular diseases and all-cause deaths [10, 11]. Urinary albumin level, urinary protein level, and estimated glomerular filtration rate (eGFR) are clinically evaluated as the markers of renal function [12, 13]. Previous studies have demonstrated that urinary albumin level [14–16] or decreased eGFR [17, 18] is related to insulin resistance, inflammation, oxidative stress, and vascular endothelial dysfunction. Interestingly, these factors have also been reported as those contributing to sarcopenia [17, 19–21], suggesting that sarcopenia may be associated with urinary albumin level, urinary protein level, and decreased eGFR.

Previous studies involving patients with diabetes have indicated that sarcopenia is related to urinary albumin level [22], urinary protein level [23], and/or decreased eGFR [24]. Investigating the association between sarcopenia and the aforementioned parameters in patients with diabetes is important considering early detection and intervention in such patients with decreased renal function. Meta-analysis allows the robust analysis of these associations. The present study investigated the association between sarcopenia and urinary albumin level, urinary protein level, and eGFR in patients with diabetes via the meta-analysis of observational studies.

## 2. Materials and Methods

### 2.1. Study Selection

A systematic review was performed in accordance with the Preferred Reporting Items for Systematic Reviews and Meta-analysis statement [25]. A literature search was performed on February 1, 2019, using MEDLINE, Cochrane Controlled Trials Registry, CINAHL, and ClinicalTrials.gov. The search strategy involved the following terms: sarcopenia (Medical Subject Heading (MeSH)), hand strength (MeSH), muscle strength (MeSH), walking speed (MeSH), sarcopeni^∗^, muscle mass, fat free mass, grip strength, or muscle power; glomerular filtration rate (MeSH), proteinuria (MeSH), albuminuria (MeSH), kidney disease (MeSH), kidney failure, renal insufficiency, chronic (MeSH), diabetic nephropathies (MeSH), urine protein, urinary albumin, kidney failure, kidney function, renal failure, renal function, nephropathy, or nephropathies; and diabetes mellitus (MeSH), diabet^∗^, IDDM, NIDDM, T1DM, T2DM, T1D, OR T2D. The inclusion criteria ensured that studies investigating the association between sarcopenia and urinary albumin level, urinary protein level, and eGFR and calculating ORs and 95% CIs were included. Reviews, letters, comments, reports on studies in animals, and duplicate literature were excluded.

We used data comparing the highest severity group with a normal group when using studies in patients stratified based on the severity of sarcopenia. We used data involving the longest duration when using studies on the same cohort. Studies published in both English and Japanese were included. Two authors (SI and RK) independently evaluated whether each report met the inclusion of the present study. In cases of differing interpretation between the two authors, two other authors (KI and KM) were consulted. Ethics approval was not applicable for this study.

### 2.2. Data Extraction and Quality Assessment

We prepared a data extraction form describing the characteristics of included studies (key author's name, publication year, study location, study design, sample size, participants' basic information, sarcopenia definition and prevalence, outcome, and adjustment factors). Continuous variables were presented as means, standard deviations, standard errors, and 95% CIs, whereas dichotomous variables were presented as percentage (%). Studies with confounders that led to optimized adjustment were included if several ORs were reported in a single study. Quality evaluation was performed using the risk of bias assessment tool for nonrandomized studies [26]. Low, moderate, and high risks of bias were used to evaluate the following six domains: patient selection, confounding variables, exposure measurements, the blinding of outcome assessors, incomplete outcome date, and selective outcome reporting.

### 2.3. Statistical Analysis

We calculated pooled OR and 95% CI of the association between sarcopenia and urinary albumin level, urinary protein level, and eGFR. OR and 95% CI were converted into natural logarithm (logOR) and standard error values. Analysis was performed using a random-effects model, and *I*^2^ was used to evaluate heterogeneity (*I*^2^ ≥ 50%: heterogeneity [27]). Subgroup analysis was used to evaluate age (≥60 vs. <60 years), the procedures of sarcopenia assessment (dual-energy X-ray absorptiometry (DXA) vs. others), eGFR (≥90 vs. <90 mL/min/1.73 m^2^ and ≥60 vs. <60 mL/min/1.73 m^2^), and sex (the proportion of women ≥ 50% vs. <50%). When ≥10 studies were included in the analysis, we constructed Funnel plots to evaluate publication bias [28]. Analysis was performed using the RevMan version 5.3 (Cochrane Collaboration, http://tech.cochrane.org/revman/download, March 2019), and the statistical significance was set at *P* < 0.05.

## 3. Results

### 3.1. Description of Included Studies and Assessment of Potential Bias

**Figure 1:**
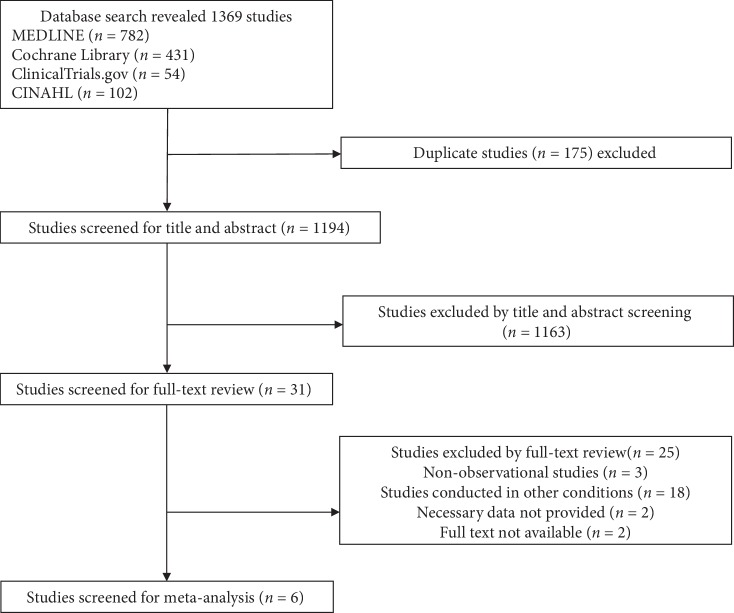
Study flow diagram.

The literature search extracted 1376 papers; of these, six studies (2662 patients) met the inclusion criteria and were included in the meta-analysis (Figure 1) [22–24, 29–31]. The characteristics of the six studies are summarized in Table 1. All the studies had a cross-sectional design except one study [30]. The mean age of patients was 60 years, and women accounted to 46.3% of all patients. One study used equations [24] to evaluate sarcopenia, and the other studies used DXA. The frequency of sarcopenia was 17.9%.

Regarding the quality of the studies included, the proportions of appropriate assessments on different domains were as follows: participant selection, 100% (6/6); confounding variables, 50% (3/6); exposure measurement, 100% (6/6); blinding of outcome assessors, 100% (6/6); incomplete data, 100% (6/6); and selective reporting, 100% (6/6) (Table 2). Bias among the included studies was attributed to confounding variables. Moreover, we did not employ Funnel plots because the number of studies included was <10.

### 3.2. Association between Sarcopenia and Urinary Albumin

**Figure 2:**
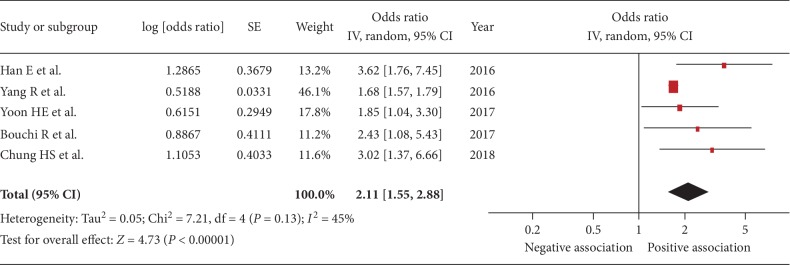
Forest plot of the association between sarcopenia and albuminuria. Odds ratios (ORs) in individual studies are presented as squares with 95% confidence intervals (CIs) presented as extending lines. Pooled OR with its 95% CI is indicated by a diamond.

**Figure 3:**
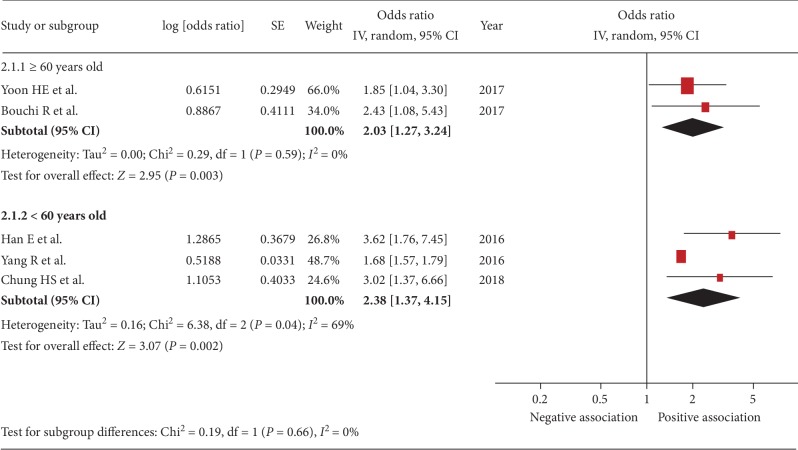
Subgroup analysis: forest plot of the association between sarcopenia and albuminuria based on age ≥ 60 or <60 years. Odds ratios (ORs) in individual studies are presented as squares with 95% confidence intervals (CIs) presented as extending lines. Pooled OR with its 95% CI is indicated by a diamond.

**Figure 4:**
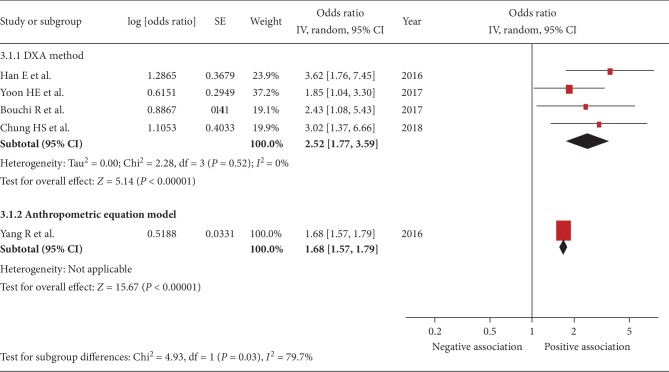
Subgroup analysis: forest plot of the association between sarcopenia and albuminuria based on the method of sarcopenia assessment. Odds ratios (ORs) in individual studies are presented as squares with 95% confidence intervals (CIs) presented as extending lines. Pooled OR with its 95% CI is indicated by a diamond.

**Figure 5:**
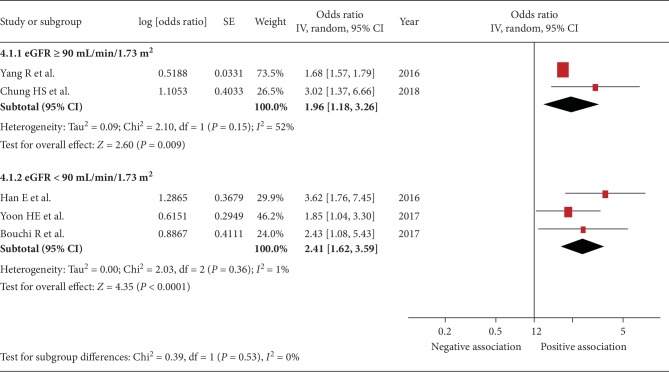
Subgroup analysis: forest plot of the association between sarcopenia and albuminuria based on eGFR ≥ 90 or <90 mL/min/1.73 m^2^. Odds ratios (ORs) in individual studies are presented as squares with 95% confidence intervals (CIs) presented as extending lines. Pooled OR with its 95% CI is indicated by a diamond.

**Figure 6:**
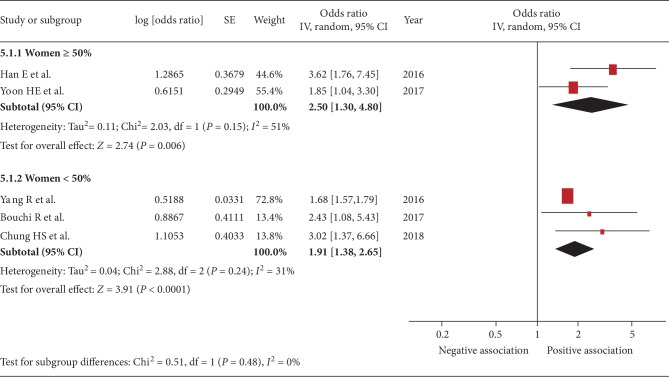
Subgroup analysis: forest plot of the associations between sarcopenia and albuminuria based on the proportion of women being ≥50% or <50%. Odds ratios (ORs) in individual studies are presented as squares with 95% confidence intervals (CIs) presented as extending lines. Pooled OR with its 95% CI is indicated by a diamond.

Five studies were included [22, 24, 29–31]. The pooled OR of the association between sarcopenia and urinary albumin level was 2.11 (95% CI, 1.55–2.88; *P* < 0.001; *I*^2^ = 45%, Figure 2), indicating a significant association. Figures 3–6 show the results of subgroup analysis. Sarcopenia was significantly associated with urinary albumin level regardless of age, the procedures of sarcopenia assessment, eGFR, and sex.

### 3.3. Association of Sarcopenia with Urinary Protein Level and eGFR

**Figure 7:**
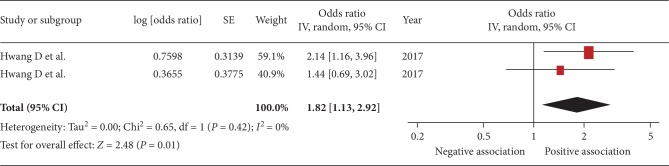
Forest plot of the association between sarcopenia and proteinuria. Odds ratios (ORs) in individual studies are presented as squares with 95% confidence intervals (CIs) presented as extending lines. Pooled OR with its 95% CI is indicated by a diamond.

**Figure 8:**
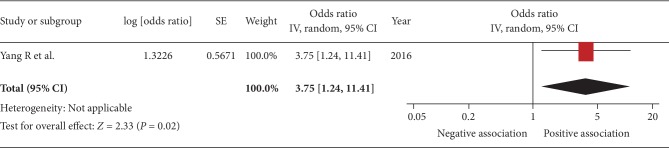
Forest plot of the association between sarcopenia and eGFR. Odds ratios (ORs) in individual studies are presented as squares with 95% confidence intervals (CIs) presented as extending lines. Pooled OR with its 95% CI is indicated by a diamond.

The pooled OR of the association between sarcopenia and urinary protein level was 1.82 (95% CI, 1.13–2.92; *P* = 0.01; *I*^2^ = 0%; Figure 7). Pooled OR of the association between sarcopenia and decreased eGFR was 3.75 (95% CI, 1.24–11.41; *P* = 0.02; Figure 8).

## 4. Discussion

The present study investigated the association between sarcopenia and urinary albumin level, urinary protein level, and eGFR in patients with diabetes via a meta-analysis of observational studies. The results demonstrated a significant association between sarcopenia and urinary albumin level. This association was also indicated by subgroup analyses involving age, the procedures of sarcopenia assessment, eGFR, and sex. Sarcopenia was also found to be associated with urinary protein level and decreased eGFR; however, these analyses were performed using few studies, leading to a lack of robustness in the results.

A previous study based on the national health survey data [29] demonstrated that OR of the association between sarcopenia and urinary albumin level was approximately 1.63–2.34, indicating a significant association. The report also suggested that diabetes is the second major factor after that contributes to urinary albumin level. Thus, diabetes and sarcopenia may be important factors associated with urinary albumin level. The results of pooled analysis in the present study, which was solely conducted on patients with diabetes, showed that the pooled OR of the association between sarcopenia and urinary albumin level was 2.11, which is consistent with the findings of a previous study [29]. These results are likely to be robust because no heterogeneity was observed in the analysis. Other studies have demonstrated the effects of sex on the association between sarcopenia and decreased renal function (urinary albumin level and the duration of kidney disease) [31, 32]. These reports demonstrated that an association between sarcopenia and decreased renal function was only observed in men; the possible cause of this is a significant decrease in testosterone and dehydroepiandrosterone level [33, 34] and a decrease in physical activity with increasing age in men [32]. Moreover, in the present study, a subgroup analysis was solely conducted on women, resulting in the absence of a sex-based analysis. Further investigation of the effects of sex on the association between sarcopenia and urinary albumin level in patients with diabetes, including the mechanism involved, is needed.

According to a previous study, the OR of the association between sarcopenia and urinary protein level [23] was 2.84. The results of the pooled analysis in the present study on patients with diabetes indicated that the pooled OR of the association between sarcopenia and urinary protein was 1.82, which was slightly lower than that found in the previous study [23]. Differences in background factors may play a role in the detection of this difference. In particular, the mean eGFR of patients in the previous study [23] was approximately 70 mL/min/1.73 m^2^ whereas that in the pooled analysis in the present study was 90 mL/min/1.73 m^2^, indicating relatively well-preserved renal function in the populations in the present study. The severity of renal function deterioration has been found to be closely associated with chronic inflammation, oxidative stress, and insulin resistance, which are also associated with sarcopenia [17, 19–21]. We surmise that the observed difference in the OR of the association between sarcopenia and urinary protein level was because the number of patients with decreased renal function was higher in the previous study than that in the present study.

The association between sarcopenia and decreased eGFR in the present study was analyzed from the data in the report by Yang et al. [24]. This report investigated patients without diabetes and found a significant association between sarcopenia and decreased eGFR in both men and women [24]. However, care should be taken in the interpretation of these associations; serum creatinine level was used to calculate eGFR in studies included in the aforementioned report; thus, a decrease in muscle mass may have led to a decrease in serum creatinine level. Therefore, we assume that this decrease in eGFR was due to a decrease in muscle mass and is not always reflected as a decrease in renal function [35]. A procedure for the evaluation of renal function that is unaffected by muscle mass includes GFR assessment using cystatin C; the usefulness of this procedure has been previously reported [36]. Further investigation regarding the association between sarcopenia and eGFR in patients with diabetes is warranted using a more precise procedure of renal function assessment.

Recently, the maintenance of renal function and the prevention of the onset of chronic kidney diseases in patients with diabetes have been significantly important challenges. Kidney diseases in patients with diabetes result in end-stage renal diseases, increased rates of the introduction of dialysis, increased mortality, and increased medical costs [10, 11, 37]. Counseling on sodium-restricted diet and other diet therapies and strictly controlling glucose level, blood pressure, and lipids are required for renoprotection in patients with diabetes [38, 39]. The results of the present study suggest that in addition to the above approaches, caution and early intervention against sarcopenia are crucial for renoprotection. Several studies have demonstrated that nutrition [40] and exercise [41] may improve sarcopenia. Urinary albumin level, urinary protein level, and decreased eGFR, which are the indices of sarcopenia and renal function, are related to many common factors such as insulin resistance, inflammation, and oxidative stress. Further investigation regarding the effects of intervention against sarcopenia on renoprotection in patients with diabetes is warranted.

This study has several limitations. First, the included literature may have involved databases that were not included in our search, and this may have affected study results. Second, some studies involving data with insufficient adjustment of confounders were included in the present analysis, which may have led to a bias. The results of the present study may be overestimated due to the insufficient adjustment of confounders, and attention should be paid to the interpretation of the result. Third, the definition of sarcopenia differed in the included studies. Differences in the methods for calculating the limb skeletal muscle mass index (SMI) and in the cutoff values used may affect the results. Fourth, the number of studies included in the meta-analysis was relatively small. Fifth, all studies included were conducted in Asia; data from other regions are also necessary for robust analysis. Lastly, since most of the studies used in the present meta-analysis were cross-sectional studies, it is difficult to refer to any causal relationship between the sarcopenia and the renal function. In the future, further study is required for integrating these cross-sectional studies.

## 5. Conclusions

In conclusion, the present study evaluated the association between sarcopenia and urinary albumin level, urinary protein level, and eGFR via the meta-analysis of studies on diabetes. The results showed a significant association between sarcopenia and urinary albumin level. The association between sarcopenia and urinary protein level and decreased eGFR was also observed, but the results were not robust as a limited number of studies were included. Further investigation is needed considering these limitations.

## Figures and Tables

**Table 1:** Characteristics of the studies included in the present meta-analysis.

No.		Reference	Year	Region	Design of study	No. of patients	Age^†^ (years)	Women (%)	HbA1c (%)	eGFR (mL/min/1.73 m^2^)	Definition of sarcopenia	Sarcopenia (%)	Outcome	Adjustment
1		Han et al. [29]	2016	Korea	Cross-sectional	360	58	56.5	NR	87	ASM/height^2^ below the cutoff value (7.0 kg/m^2^ in men and 5.4 kg/m^2^ in women) using the DXA method according to the recommendations of AWGS	18.8	Albuminuria	HTN, BMI, and MetS
2		Yang et al. [24]	2016	China	Cross-sectional	793	51	30.6	9.2	114	ASM/height^2^ below the cutoff value (7.26 kg/m^2^ in men and 5.45 kg/m^2^ in women) using the anthropometric equation model	26.2	Albuminuria and eGFR	Age, BMI, SBP, DBP, HbA1c, FPG, diabetes duration, smoking status, alcohol drinking status, the use of drugs, and physical activities
3		Bouchi et al. [30]	2017	Japan	Longitudinal study	238	64	39.2	7.1	75.6	SMI (ASM/height^2^) below the cutoff value (7.0 kg/m^2^ in men and 5.4 kg/m^2^ in women) using the DXA method and grip strength below the cutoff value (26 kg in men and 18 kg in women) according to the recommendations of AWGS	17.6	Albuminuria	Age, sex, HbA1c, BMI, and TG/HDL-C ratio
4		Hwang et al. [23]	2017	Korea	Cross-sectional	704	69	49.5	NR	78.1	SMI (ASM/weight) of 2 SD below the sex-specific mean value for a younger reference group (cutoff point for sarcopenia in men and women: 27.2% and 21.3%, respectively)	14.2	Proteinuria	None
5		Yoon et al. [31]	2017	Korea	Cross-sectional	158	64	54.7	NR	87.6	SMI (ASM/weight) of 2 SD below the sex-specific mean value for a younger reference group	11.3	Albuminuria	Age, BMI, smoking status, alcohol drinking status, physical activities, HTN, DL, CVD, MetS, vitamin D deficiency, estrogen replacement, and renal dysfunction (eGFR < 60 mL/min/1.73 m^2^)
6		Chung et al. [22]	2018	Korea	Cross-sectional	409	58	47.4	7.1	102.8	SMI (ASM/weight) of 2 SD below the sex-specific mean value for a younger reference group (cutoff point for sarcopenia in men and women: 35.9% and 30.6%, respectively)	19.6	Albuminuria	Sex, age, percent body fat, smoking status, alcohol status, physical activity, duration of diabetes, HbA1c, SBP, LDL cholesterol, HDL cholesterol, triglyceride, and RAS blocker, statin, fibrate, and insulin use

^†^Unless indicated otherwise, data are shown as mean values. Abbreviations: SD: standard deviation; SMI: skeletal muscle mass index; BMI: body mass index; DXA: dual-energy X-ray absorptiometry; ASM: appendicular skeletal muscle mass; AWGS: Asian Working Group for Sarcopenia; HTN: hypertension; MetS: metabolic syndrome; DL: dyslipidemia; SBP: systolic blood pressure; DBP: diastolic blood pressure; TG: triglycerides; LDL: low-density lipoprotein; HDL-C: high-density lipoprotein-cholesterol; CVD: cardiovascular disease; HbA1c: hemoglobin A1c; eGFR: estimated glomerular filtration rate; RAS: renin-angiotensin system; NR: not reported.

**Table 2:** Risk of bias assessment included in the meta-analysis.

No.	Reference	Selection of participants	Confounding variables	Measurement of exposure	Blinding of outcome assessment	Incomplete outcome date	Selective outcome reporting
1	Han et al. [29]	L	H	L	L	L	L
2	Yang et al. [24]	L	L	L	L	L	L
3	Bouchi et al. [30]	L	H	L	L	L	L
4	Hwang et al. [23]	L	L	L	L	L	L
5	Yoon et al. [31]	L	L	L	L	L	L
6	Chung et al. [22]	L	H	L	L	L	L

Abbreviations: L: low risk of bias; U: unclear risk of bias; H: high risk of bias.

## Data Availability

The data that support the findings of this study are available from the corresponding author upon reasonable request.
